# Sequence context affects the rate of short insertions and deletions in flies and primates

**DOI:** 10.1186/gb-2008-9-2-r37

**Published:** 2008-02-21

**Authors:** Amos Tanay, Eric D Siggia

**Affiliations:** 1 Center for Studies in Physics and Biology, The Rockefeller University, York Ave, New York, NY 10021, USA; 2 Department of Computer Science and Applied Mathematics, Weizmann Institute of Science, Rehovot, Israel 76100

## Abstract

**Background:**

Insertions and deletions (indels) are an important evolutionary force, making the evolutionary process more efficient and flexible by copying and removing genomic fragments of various lengths instead of rediscovering them by point mutations. As a mutational process, indels are known to be more active in specific sequences (like micro-satellites) but not much is known about the more general and mechanistic effect of sequence context on the insertion and deletion susceptibility of genomic loci.

**Results:**

Here we analyze a large collection of high confidence short insertions and deletions in primates and flies, revealing extensive correlations between sequence context and indel rates and building principled models for predicting these rates from sequence. According to our results, the rate of insertion or deletion of specific lengths can vary by more than 100-fold, depending on the surrounding sequence. These mutational biases can strongly influence the composition of the genome and the rate at which particular sequences appear. We exemplify this by showing how degenerate loci in human exons are selected to reduce their frame shifting indel propensity.

**Conclusion:**

Insertions and deletions are strongly affected by sequence context. Consequentially, genomes must adapt to significant variation in the mutational input at indel-prone and indel-immune loci.

## Abstract 2

Analysis of a large collection of short insertions and deletions in primates and flies shows that the rate of insertions or deletions of specific lengths can vary by more than 100 fold, depending on the surrounding sequence.

## Background

The evolution of genomes is driven by an influx of mutations that are subject to a stochastic process of neutral fixation and to multiple selective pressures that can change the neutral fixation dynamics. Good understanding of the evolutionary process requires characterization of both the mutational and fixation processes. This is particularly important in applications that try to reveal genomic loci that are evolving under selection by looking for slowly or rapidly evolving sequences. In such studies one has to make sure the mutational input at the genomic regions under study is not abnormally high or low [1-4], or else the inferred selection may be an artifact of the mutational dynamics and not a true indication for a functional constraint on the sequence. Changes are introduced into genomes through point mutations, insertions and deletions. The dynamics of each of these mechanisms may vary according to genomic context and the presence of various factors acting in *trans*.

Before the availability of numerous fully sequenced genomes, evolutionary studies focused on two extremes: replacements of entire genes and chromosome domains or point mutations. The former can be detected over long evolutionary times and their gain or loss has an immediate functional interpretation. Quantitative molecular evolution has developed around the occurrence of point mutations over limited regions of the genome, where it is feasible to compare intra-species variation with inter-species change, and infer fitness. Intermediate in scale are small (1-50 bp) insertions or deletions (indels). They are less numerous than single base substitutions, but can account for comparable base-pairs of change. For example, 3.2% of the base-pair changes between the fly species and 0.8% of the base-pair changes in the primate species analyzed here are affected by indels, compared with 1.8% and 1.5% affected by point mutations in flies and primates, respectively. Short indels are, therefore, a significant factor in the mutational input that feeds into the evolutionary process, a fact that underlines the importance of characterizing the mechanisms that induce or suppress their activity. Earlier work focused on human insertions and deletions at disease loci [5-8] or on indels detected between relatively distant species [9,10] suggested that such events are correlated with specific sequence contexts. More recent works [11,12] characterized extensive collections of indels in the human-chimp lineages, further motivating a comprehensive approach to the description of their sequence contexts.

In this work, we construct an evolutionary model for small indels in flies and primate genomes. We characterize these processes using mechanistic insights (tandem duplication for insertion, replication slippage for deletion). We discover significant sequence contexts that are susceptible to deletion or insertion. Using the new data, we are able to predict the rate of insertions and deletions at each genomic loci given the sequence surrounding it. We show the indel rate at different loci can vary within more than two orders of magnitude, making specific loci susceptible to rapid insertion or deletion and other loci immune to it. Our results suggest that indels are introduced into the genome by a random process, but that the rate of this random process is highly dependent and, to a great extent, predictable from the sequence. We demonstrate the significance of this indel rate variability by showing how synonymous codons in human exons are selected for low frame shifting indel potential.

## Results

### A comprehensive compendium of short insertion/deletion events in primates and flies

Close, fully sequenced species grouped around one species with high quality annotation permit good single indel event statistics to be inferred. Three species are necessary, the two closest 'ingroups' are compared, while the third 'outgroup' defines the ancestor and thus distinguishes insertions from deletions. For the primates we compared human and chimpanzee with *Rhesus macaque *as the outgroup. Human indels inferred from these three species have recently been studied by Messer and Arndt [12] and using a non-primate outgroup by Chen *et al*. [11]. For flies, we compared the *Drosophila *species *D. simulans *with *D. sechelia*, using *D. melanogaster *as the outgroup, refining an earlier study [10] that compared *D. melanogastar *with *D. yakuba *using *D. pseudoobscura *as the outgroup.

**Figure 1:**
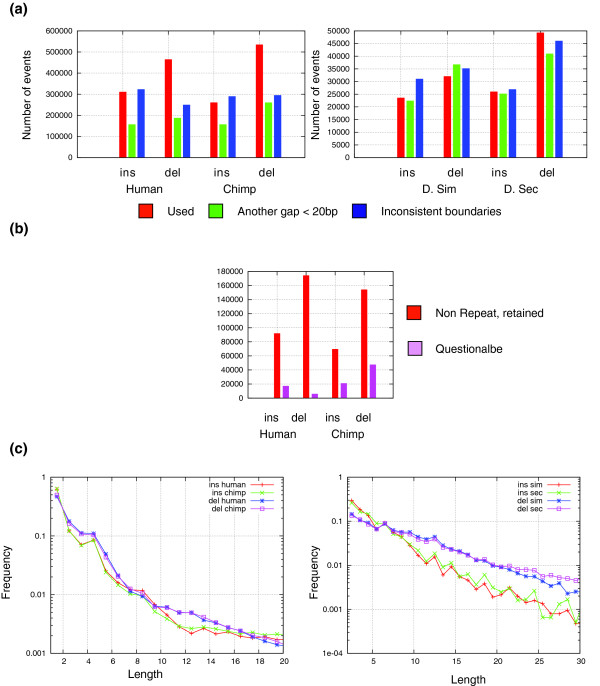
A compendium of insertions and deletions in primates and flies. **(a) **Gaps and their boundaries. The bar charts represent the total number of insertions (Ins) and deletions (del) at each lineage, resolved using the known phylogenetic relations between the species. Gaps with ambiguous boundaries or flanking gapless matches of less than 20 bp were filtered out since they either represented superimposed events or alignment problems. D. Sec, *D. sechelia*; D. Sim, *D. simulans*. **(b) **Filtering questionable indels. Shown are the numbers of non-repetitive primate indels that were retained or filtered as questionable based on direct genomic searches (see Materials and methods). **(c) **Insertion and deletion length distributions. Shown are the distributions of insertion and deletion lengths in our primate and fly compendia. Graphs are drawn in log-scale. A non-geometric trend is apparent in the primate and fly insertion data and in the primate deletion data (the probability of a single exponential fit has *P *< 10^-150 ^(KS goodness of fit)). The peaks at, for example, primate insertions of length 4 and 8 are not understood but statistically significant. The fluctuation around the trend for longer events reflects lower sample sizes.

Our aim is to model the indel rates from the sequence context. We therefore developed a filtering and weighting scheme in an attempt to extract a maximal amount of insertion and deletion loci for which the sequence context is unambiguous. One class of potentially ambiguous indels consists of loci that were affected by multiple insertion or deletion events. Multiple events may result in inconsistent gap boundaries among the aligned species. Interestingly, although the global rate of indels in the phylogenies we have analyzed was less than 1 event per100 bp, 33% of the gaps in the primate alignments (35% in the flyalignments) had inconsistent boundaries. To avoid ambiguities in sequence context, we filtered out such gaps from further analysis and, for similar reasons, we also filtered gaps that had another gap within 20 bp of the insertion or deletion point. To account for gaps with ambiguous positions, we determined all possible equally probable alignments of each gap and treated them uniformly (see Materials and methods). Statistics on gaps and inferred insertions and deletions are provided in Figure 1a.

We further controlled for possible alignment problems in primates by performing direct searches for putative human and chimp inserts and their flanking 60 bp in the chimp and human genomes, respectively (see Materials and methods). We performed similar searches for the sequences flanking putative deletion points. All putative insertion or deletion sequences (including the flanking regions) that were aligned to the other genome without gaps were defined as questionable and removed from further analysis (Figure 1b). Comparison of two multiple alignment sets (based on the panTro1 and panTro2 assemblies) revealed that many of the questionable indels are inconsistent between versions of the alignment (data not shown). On the other hand, analysis of sequence quality data in the chimp assembly did not support a connection between dubious indels and low sequence quality.

### Distribution of inserted and deleted sequence lengths

It is widely assumed (for example, by alignment algorithms) that gap lengths are distributed as geometric variables, but as shown in Figure 1c the length distributions of the events in our set are not geometric, and vary between insertions and deletions. In the primate lineages, the length distributions for both insertions and deletions may reflect two geometric regimes, making short and long events more probable than expected given a simple, single parameter geometric distribution. The shift between the two regimes occurs at length 8-10 bp for deletions and at length 10-12 for insertions. For flies, the deletion lengths are distributed as a simple geometric distribution. The insertions in flies are also distributed with two geometric regimes, one for short events (2-10 bp) and the other for longer ones (10 bp and more). The observed length distributions can indicate that multiple mechanisms are contributing to the insertion or deletion processes. Interestingly, the length distributions of questionable events are markedly different than those of the retained events, supporting our filtering scheme (Figure S1 in Additional data file 1). We were unable to detect specific families of sequences that significantly affect the length distribution in any of the tested lineages. Previous studies that argued for geometric gap length distributions [13-15] were based on smaller numbers of events than present in our set, and were, therefore, limited with respect to inference of the distribution of low frequency (that is, long) events (which are those that seem to break the simple geometric regime).

### Most short insertions are accounted for by simple and complex tandem duplications, sometimes involving the reverse strand

**Figure 2:**
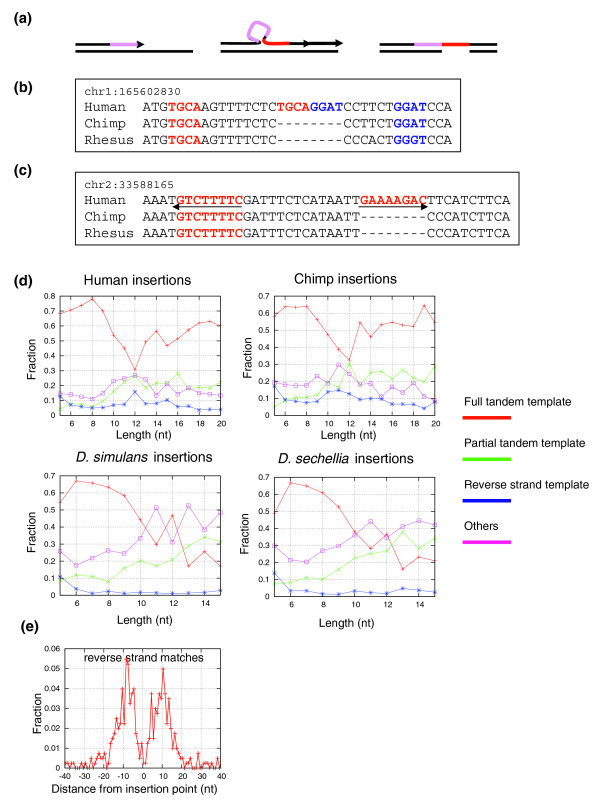
Perfect and complex short tandem duplications in primates and flies. **(a) **Exact tandem duplication. Replication errors following scenarios similar to the illustrated scheme will generate insertions that are flanked by their exact template. **(b) **Complex duplications. The 8 bp insertion illustrated lacks a simple tandem template, but can be divided into two parts, each of which have a perfect match in proximity to the insertion point **(c) **Reverse strand templates. The 8 bp insertion illustrated lacks a perfect or partial template in the vicinity of the insertion site. It does have a perfect template at the reverse strand, however, located 14 bp from the insertion point. **(d) **Fraction of insertions with perfect, partial and reverse template matches. Shown are the fractions of insertions that admitted a full tandem template, partial tandem template or reverse strand template, plotted as a function of the insertion length (see Materials and methods). Most of the remaining events (designated 'other') are likely to be artifacts (see text). Nt, nucleotides. **(e) **Spatial distribution of reverse strand templates. Shown is the distribution of relative positions of reverse strand templates for human/chimp insertions. The distribution was computed using insertions of at least 6 bp that lacked a tandem template in the plus strand but admitted a perfect reverse strand template.

Short insertion events were shown before to be mechanistically possible through micro-tandem duplication (illustrated in Figure 2a) [16]. We found that the majority, but not all, of the insertions in our compendium have a tandem match (or 'template'), suggesting that simple tandem duplication is the major mechanism for short insertions and confirming earlier results [5,6,12]. To further characterize insertions that lack a good tandem match, we studied the matching between subsets of the inserted sequences and their surrounding sequences, carefully controlling for spurious regional sequence matches, hypothesizing that several copying events might explain these insertions (Figure 2b; see Materials and methods), as previously suggested for indels associated with human disease loci [8]. We also tested the matching between inserted sequences and the proximal sequence of the reverse strand, following observations of inserts with perfect reverse strand templates (Figure 2c; see Materials and methods). Using data on all the insertions in both our compendia (Figure 2d), we could attribute about 80-90% of the insertions to a simple or complex tandem duplication event. Complex events become more prominent for longer insertions. We observed a considerable number of primate events with a template on the reverse strand. A number of cases in bacteria have been documented where DNA polymerase can transiently switch strands at the replication fork [17,18], although these did not involve more than a single base indel. We further analyzed the distances between the reverse complement insert and the presumed original sequence (Figure 2e). There is a pronounced peak of reverse templates located about 10 bp from the insertion point, confirming the non-randomness of the reverse strand templates and suggestive of helical phasing.

The remaining putative insertions lack apparent sequence templates in their immediate neighborhood. To see if sequence templates for such insertions can be found in more remote chromosomal regions, we examined insertion events of length above 30 bp (for which genome-wide searches are specific) that lacked a tandem template. We detected only few cases where a possible insert template was located out of the immediate locus neighborhood. In no case did we find a possible insert template in a different chromosome. We next computed the average chimp assembly (panTro2) quality around putative insertion events with and without a tandem template. We could not detect a significant difference in the sequence quality around the two groups (Figure S2 in Additional data file 1). In flies we found that the many non-tandem long inserts were present (though mutated) in *D. yakuba*. This suggests another instance of incomplete lineage sorting [19], where structurally polymorphic loci persist throughout the speciation of the *Drosophila *species we analyzed. Other effects (for example, alignment artifacts) may also be contributing to the increase in non-tandem fly insertion fraction as a function of the insert length. To summarize, for our compendium, the dominant mechanism for insertions is tandem duplication, perhaps in several steps, and in a minority of cases the copy is from the complementary strand. A fraction of the gaps still cannot be rationalized using this model, and although there is some indirect evidence that suggest many of these are in fact alignment errors, other explanations are still possible.

### Sequence preferences of short insertion events

**Figure 3:**
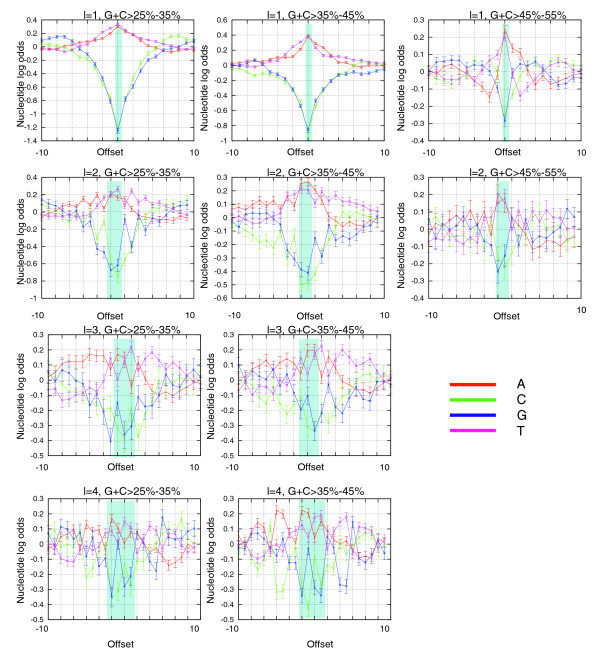
Sequence preferences around human insertions. Shown are profiles of log odds (base 2) for the nucleotide composition around human insertions of lengths 1-4. The 0.95 confidence intervals are plotted for each frequency. For each offset relative to the insertion point, we computed the frequency of each nucleotide and compared it to the frequency in genomic regions with similar GC content. The graphs indicate strong preferences for specific nucleotides in the context of an insertion, suggesting these nucleotides are contributing to the insertion process. The shaded region represents the nucleotide profile of the inserted sequence itself. Profiles around chimp insertions are highly similar to those shown here (Figure S3 in Additional data file 1). The number of events in flies is too small to reliably define these profiles.

While tandem duplications explain the majority of short insertions in all lineages we considered, we wished to explore possible contribution of specific sequence contexts to the initiation of the duplication process. It is known, for example, that even imperfect stems can stabilize sequence intermediates that enhance rearrangements [20]. We wished to generalize and quantify this phenomenon using our compendium of short insertion events. We first built a profile model for each insertion length by computing the nucleotide frequencies around insertion points. Working with large genomes (and many insertion events), the profiles were very robust statistically. Moreover, depending on the event length, the profiles proved informative, indicating many specific nucleotide preferences that deviate from the expected background pattern. To control for the non-uniform distribution of nucleotides in genomes (and the human genome in particular), we next recomputed the profiles for groups of insertion events that are present in regions with predefined G+C content, and used the background nucleotide distribution in such regions to compute a log odds score for each profile entry (see Materials and methods; Figure 3). The results reflect several universal preferences and other preferences that are particular to specific insertion lengths. Overall, insertion points are flanked by low GC content (when compared to the regional GC content), with more A than T nucleotides towards the 5' end and more T than A nucleotides towards the 3' end. It is possible that such contexts make the replication process more vulnerable to slippage and reconnection, or affect the capacity of the sequence to form short loops [17,20]. As we shall see below, we can use the identified sequence preferences to predict the rate of insertions at each genomic locus, although their mechanistic role awaits further characterization.

### Short deletions are marked by short matches at the deletion junction

**Figure 4:**
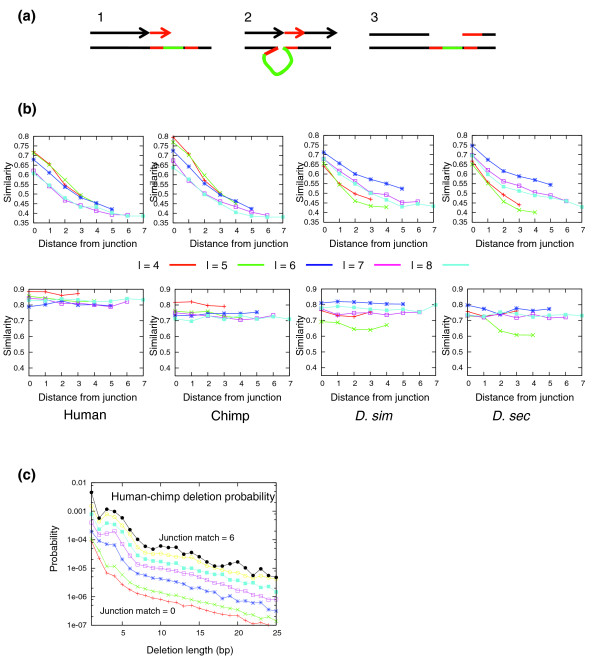
Short matches define the limits of a deletion. **(a) **Deletion via replication slippage. Illustrated is a process by which a replication fork slips from the sequence and reconnects at a different locus, thereby deleting the short sequence shown. The stability of a slippage event is controlled by the length of matching sequence (red) at the deletion junction (arrow) **(b) **Similarity between deleted/inserted sequence and its flanking nucleotides. We computed the similarity between deleted (first row) and inserted (second row) nucleotides and the nucleotides flanking them (comparing each position to the position l nucleotides away, where l is the indel length). We used the flanking sequence in the side with better overall percent identity and averaged the statistics over all optimal alignments to control for alignment algorithm artifacts (see Materials and methods). For insertions we observe high similarity that is unaffected by the distance from the junction, while for deletions the similarity is rapidly decreasing as a function of the distance. D. Sec, *D. sechelia*; D. Sim, *D. simulans*. **(c) **Deletion rates. Shown are deletion rates, as a function of the deletion length, for various junction match lengths (denoted s') from 0 (bottom) to 6 (top). The rates are normalized by the background genomic frequency of identical sequences of length s spaced by l bps. The rate increases by a factor of 100-fold with s, but retains the same slope, regardless of the deletion length l. Combined human and chimp data are shown.

Figure 4a illustrates a possible mechanism for deletion of short sequences during replication. Sequences are lost after slippage of the replication machinery at one locus, followed by re-association with the DNA at a different locus, leaving the sequence between the two loci unreplicated. It is likely that some similarity between the sequences of the slippage and re-association loci would contribute to the deletion process [6,7]. To quantify this effect, we computed the percent identity between deleted sequences and their immediately flanking sequences, and compared it to the percent identity of inserted sequences and their tandem sequences (see Materials and methods). We used the flanking sequence on the side that had better overall percent identity. As expected, we derived contrasting results for deletions and insertions (Figure 4b). The percent identity for insertions is high and stable along the entire inserted sequence, agreeing with the statistics on tandem duplications discussed above. For deletions, we observed high similarity for the first few base-pairs at the deletion junction, but then rapid decrease in similarity to the expected background levels. Similar results are observed for flies and primates. These results support the mechanism outlined above and confirm that replication slippage can be induced by matching of few base-pairs around the junction. To further quantify this effect, we compared the number of deletion events of length l and deletion junction matching of length s to the overall number of genomic loci with s matching base-pairs spaced by l nucleotides. The ratio between these numbers reflects the probability of deletion given a deletion junction match of length s. As shown in Figure 4c, the probability of deletion increases by a factor of 100 between loci without any match at the deletion junction to loci that have at least 5 bp matching. We observed no correlation between the size of deletion and the quality of the match at the deletion junction.

### Additional sequence preferences for short deletions

**Figure 5:**
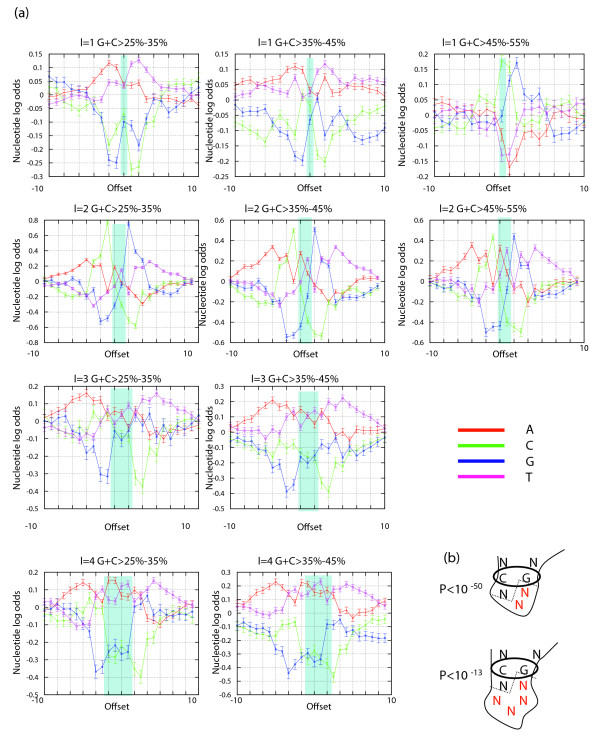
Sequence preferences around human deletions. **(a) **Nucleotide profiles. Shown are profiles of log odds for the nucleotide composition around human deletions of lengths 1-4 (Figure 3; see Materials and methods). The 0.95 confidence intervals are plotted for each frequency. For each offset relative to the deletion point, we computed the frequency of each nucleotide and compared it to the frequency in genomic regions with similar GC content. Shaded regions represent the nucleotide preferences of the deleted sequence itself. The analogous chimp profiles are shown in Figure S4 in Additional data file 1. **(b) **Higher order nucleotide correlation. Shown are schematic illustrations of the two strongest statistical associations between pairs of nucleotides flanking insertions or deletions of a specific length. Both cases represent co-occurrence of G-C pairs flanking a deletion of even size (red nucleotides represent the deleted fragment). It is possible that base pairing between these two positions contributes to specific deletion scenarios.

As is the case for insertions, deletions can be shown to have specific sequence context preferences beyond the matching of 2-3 bp at their junction. This is demonstrated by the log odds for nucleotide preferences at different GC content regions and different deletion lengths (Figure 5; computed as for insertions, see Materials and methods). First, as observed for insertions, we detect an A-T asymmetry around the deleted sequences (more As to the 5' end, more Ts to the 3' end). Unlike insertions, we also observed marked differences between Gs and Cs, where Cs are preferred just before the deletion junction and Gs after it. Many other details can be extracted from the nucleotide preference graphs, and these will be used below to construct a probabilistic model for predicting indel propensity.

We next wished to test if higher level interactions between nucleotides are significantly associated with deletion or insertions events. We searched for such interactions systematically (see Materials and methods), identifying all pairs of positions relative to the deletion/insertion sequence in which the joint distribution of nucleotide pairs differ significantly from the genomic distribution of nucleotide pairs at the same distance. The strongest pairs other than the tandem effects we discussed above (compare Figure 4b; correlation between nucleotides spaces by the length of the deletion) indicated interaction between Cs and Gs in the positions adjacent to deletions of even lengths (Figure 5b). It is possible that some sort of G-C pairing at these positions contributes to the generation and stabilization of non-B-DNA structures and, therefore, enhances deletion propensity [20]. In bacteria, palindromic sequence favors deletions since it stabilizes the fold-back configuration by internal base pairing [17].

### The indel propensity model

**Figure 6:**
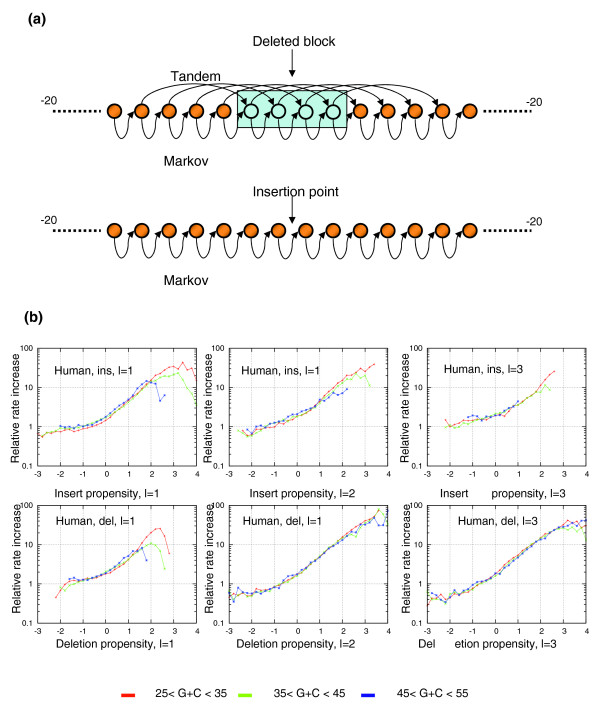
Predicting insertion and deletion rates using the indel propensity probabilistic score. **(a) **The model. We trained a positional Markov model that computes the probability of a nucleotide at each position relative to the insertion/deletion point given its position, the previous nucleotide, and possibly the nucleotide l bp upstream of it (l being the event length). Model parameterization was done separately for each length, event type (insertion/deletion) and GC content region. We can score a locus for indel propensity by dividing the probability from the model by that of a similar model estimated from background sequences. **(b) **Predicting indel rates. The graphs summarize the results of a cross validation assay consisting of training indel propensity models on half of the aligned human chromosomes and applying them to predict insertions and deletions of various lengths in the other half of the genome. For each type of event, we show the relative increase in indel probability in the human lineage as a function of the propensity score. In all cases, the model is robustly predicting an increase of 100-1,000-fold in the indel rate for high versus low scoring loci. Similar results for chimp events are shown in Figure S5 in Additional data file 1.

The sequence contexts we described above were next used to construct a probabilistic model for predicting the insertion and deletion potential at a genomic locus given its sequence context. The indel propensity models (one for each length, lineage and event type) are designed to predict the insertion (deletion) probability at a given genomic locus and phylogenetic branch (Figure 6a). The prediction is based on the sequence surrounding the locus as present in the genome prior to the putative insertion or deletion. The key features of the models are the frequencies of nucleotides at each position relative to the putative indel locus (Figures 3 and 5) and the conditional probability of nucleotides given their previous (5') nucleotide. For deletions, we also used the joint distribution of nucleotides that are spaced l nucleotides from each other (these are not used for insertions since we are applying the model to the sequence present prior to the insertion). To compute the insertion or deletion propensity for length l, we compared the likelihood of the above model to the likelihood of a similar model that was trained using the genomic background, generating a log odds score that is used for downstream analysis.

### Over 100-fold change in insertion/deletion probability given preferred sequence contexts

To test the predictive power of the models described above and to ensure the context model does not introduce overfitting, we performed standard cross validation. We divided the human genome into two (odd and even numbered chromosomes). We trained our models using the data from only odd-numbered chromosomes. We then computed the distribution of model scores for each type of event (length, lineage, type) for the background genomic sequence and around indel events occurring at even-numbered chromosomes (Figure 6b). Working with the highly similar primate genomes, we assumed the ancestral sequence is identical to the human genome (that is, we ignored point mutations), with the exception that inserted sequence has to be removed, and deleted sequence has to be retained. Cross validation confirmed the robustness of the indel propensity score, showing increasing indel probability for higher log odds values. The results indicate that the relative rate of insertions and deletions of all tested lengths vary by as much as a factor of 100 as a function of the model score. Loci with very high indel propensity have a very high probability for insertion or deletion (of a specific length) and behave much like micro-satellites. Loci with very low indel propensity may be almost indel free. Between these two extremes we observe a whole array of weaker phenomena, consisting of diverse sequence contexts with variable indel propensity.

### Indel constraints in coding regions

**Figure 7:**
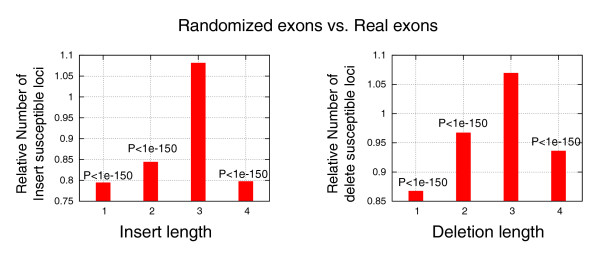
Human exons are optimized for low indel propensity. The fraction of positions with indel propensity score above 1 (indel-susceptible loci) was computed across all exons in the human genome. The graphs show the ratio between these fractions and the fraction of such loci in exons that were randomized by shuffling (preserving GC content) synonymous codons. We see a clear preference of synonymous codons for low indel propensity, except for indels of size 3, which do not cause frame shifts.

To further validate our conclusions on the importance of sequence contexts to the indel process, and to illustrate their possible functional and evolutionary significance, we analyzed the indel potential of human exons. Exons are typically under purifying selection through the proteins they encode. Degenerate codon positions are also under some secondary, multifaceted selection (for example, [21,22]) which results in the codon bias phenomenon. As demonstrated before [11], insertion and deletions in exons are likely to have particularly deleterious effects, especially when introducing a frame shift (that is when their length is not a multiple of three). We therefore hypothesized that genomes will use some of the flexibility inherent in degenerate codons to lower the indel potential of exons. To test if this is indeed the case, we compared human exons to randomized exons obtained by shuffling nucleotides in synonymous positions while maintaining the resulting amino acid sequence and regional GC content. For each insertion and deletion length, we computed the distribution of model scores in the two sets, adding up data from all loci in all human exons. As shown in Figure 7, the results reflect a significantly smaller number of coding loci with high indel propensity than what should have been expected from random selection of codons. Interestingly, this trend is highly significant (*P *< 10^-150^) for all event lengths except for 3 bp, showing that degenerate codon positions evolved to reduce the indel propensity of frame shifting indels more than frame conserving mutations. The low rate of frame shifting indels that was demonstrated before is, therefore, partially enabled by fine tuned sequence contexts, suggesting that genomes may use their sequence itself to adapt their mutability in specific functional regions.

## Discussion

The existence of a dense web of fully sequenced metazoan genomes around those of human and fly permits a thorough study of short indel events. Although their rate is below that of base substitution events, they account for a comparable number of base-pairs of sequence change and, thus, are potentially at least as important for evolving new functionality as are single base changes. The indel rate is also very context dependent, as is most immediately evident from our finding that half of the indels present between our ingroup species did not map exactly onto the outgroup. We believe this reflects a large number of multiple events that have occurred even though the primary events are rare (occurring on < 0.5% of the genome) for the genomes we compare.

New inserted sequence can usually be rationalized as a copy of adjacent sequence, but the copying process is sometimes complex or involves the complementary strand in primates. We found no systematic evidence for copying from far away (for example, 10 kb) in the size range of 50 bp or less. In the fly a possible explanation for many of the putative insertions that were not copied is incomplete lineage assortment, but this was not observed in primates. We note that there is a tradeoff to be made when choosing the outgroup: one too removed allows for multiple events and overlaid point mutations, and one too close and the phylogeny of the locus may not conform to that of the species, as is the case for some of the fly loci.

Deletions have fewer sequence constraints, namely only the propensity for the match of a few base-pairs flanking the junction (Figure 4). The relative rates of insertions to deletions differs between flies and primates for lengths over 10 bp, perhaps because most of the fly sequence is under some functional constraint [10,23], whereas the primate genomes are much less constrained. The length spectra of both insertions and deletions have two power law regimes, which *prima facie *contradicts the scoring assumption of standard alignment algorithms.

One of the emerging themes from recent analyses of divergence in closely related genomes is that the mutational process is highly dependent on sequence context. Adjacent nucleotides are known to affect point mutation rates [24] but understanding of more complex sequence context and their possible roles in changing the mutational input and/or selective pressure are only now beginning to emerge (for example, as in the case of CpG dinucleotides [25,26]. We have shown that a model using only the distribution of nucleotides suitably defined with respect to the indel predicts over 100-fold variation in the rates of the appropriate indel event. Interestingly, the sequence context of insertions and deletions of different lengths share only some of their features (for example, AT asymmetry) and differ in others (G-C coupling in deletions of even length), suggesting specific interaction with the replication machinery.

For molecular evolution, our indel propensity model can be used as a refined neutral standard in applications that search for categories of sequence that are evolving slowly due to putative selection. In particular, we have demonstrated how coding regions bias their codon usage to suppress frame shifting mutations. Indels may contribute more to the evolution of regulatory sequences than their frequency would suggest, because their size is comparable to a protein binding site. It would be interesting to see whether our model can explain some of the drift in binding sites that have been mapped on a genome scale [27-29].

## Materials and methods

### Alignments, indel detection and filtering

Primate sequences were downloaded from the remarkably useful UCSC genome browser site [30], using the hg18, panTro2 and rheMac2 assemblies. Multiple alignments were generated by extracting the primate sequences from the vertebrate maf files (28 vertebrate species, 2007 version) and concatenating contiguous fragments. To annotate known repeat sequences, we used the RepeatMasker data from the UCSC genome browser site.

We used release 4.3 of the *D. melanogaster *genome from [31]. The *D. simulans *sequence was the 'mosaic' assembly from [32], which was generated using their genome assembler to combine the sequences from six *D. simulans *strains (this assembly is also used on the UCSC browser). The *D. sechellia *sequence is a contig library from the same source. Three way alignments were done with the TBA codes from [33], and the parameters T = 1, C = 2, and L = 10,000 were modified from their defaults.

We observed for both our fly ingroup species that our alignments predicted that about 6% of all exons had a 1 bp indel when compared with the *D. melanogaster *annotation. This is an unreasonably high rate of frame shifts, so we downloaded the alignments of the individual *D. simulans *strains against *D. melanlogaster *from [34]. The six strains together with *D. melanogaster *where then multi-aligned using TBA and a new consensus was derived using the majority pattern among the *D*. *simulans *strains if one existed; otherwise, the strain matching *D*. *melanogaster *if one existed; otherwise, an array of 'N's.

With the new consensus the number of length 1 indels in exons fell to 0.4%, with the majority of these in regions of low coverage or homopolymer repeats. However, we continued to use the 'mosaic' alignment since it was no worse than the *D*. *sechellia *data, which we had no means to correct, and also the errors for indels larger than length 1 were tolerable (12% of length 2 indels and 0.5% of length 4 and larger could be spurious, estimated from the number of frame shifting indels observed (and assuming all of these are spurious) and multiplying by the fraction of coding sequence in the genome and the total number of indels we found).

To construct a reliable compendium of short insertions and deletions, we identified all gaps in the multiple alignments that had clearly defined boundaries in all three species (the outgroup aligned exactly with one of the ingroups) and were flanked by at least 20 bps of gapless matches. We annotated each gap as an insertion or deletion based on the known triplet phylogeny of primates and flies. We ignored cases that occurred in the outgroup lineage (and therefore could not be resolved as insertion or deletions). To control for arbitrary gap positions in the multiple alignments, we computed for each gap the set of all possible optimally scoring gap positions by sliding the gap in both the 5' and 3' directions, and computing the number of resulting mismatches at each position. All gap positions with the minimal number of mismatches were considered as candidates, and were assigned with a weight of 1/(Number of optimal gap positions). We used the weights when computing statistics for the insertion/deletion ensembles, unless otherwise noted. To prevent a bias from large families of short repeats, we filtered out all gaps that were within 40 bp of an annotated repeat in all the analyses reported, except for the data in Figure 1a. Including the repeats in the analysis did not affect the results significantly, nor did imposing a minimal percent identity on the ungapped flanking sequence.

To minimize alignment errors for the primate data, we further filtered events using direct genomic searches. For each insertion event we used the sequence of the insert flanked by 60 bp on each side. For deletions we used the flanking 60 bp around the deletion point. We then used J Kent's blat program (with standard parameters, see UCSC website [30]) to search for these sequences in the orthologous chromosome (searching the chimp genome for human indels and the human genome for chimp indels). We defined an event as questionable whenever blat returned a hit that spanned the insertion or deletion junction with 20 bp of flanking gapless match. The size distributions of retained and questionable events are shown in Figure S1 in Additional data file 1.

To characterize the sequence quality around different classes of indels, we used data from the chimp panTro2 assembly, extracted through the UCSC database. To generate supplementary Figure 2, we computed for each human insertion the minimal sequence quality (scaled from 0 to 97) in the aligned chimp sequence that surrounded the putative insertion point (20 bp for each side). Note that we did not use the sequence quality in the filtering process.

### Looking for sources of inserted sequences

To identify possible sequence templates for short inserts, we applied several layers of analysis. First, we directly tested the number of mismatches between the insert and its immediately flanking sequence (on both sides). In cases where more than one alignment configuration was possible for the gap, we tested all possible configurations. Inserts that had at most one mismatch for the sequence in either side were considered as perfect tandems (this was feasible for indels of length more than 5). At the second level, we searched for the longest perfect match between a substring of the insert and all sequence within 'l + 10' nucleotides on either side of the insert (of length l). We compared this number to the longest match in the 1.1 kb upstream and downstream of the insert (excluding the 100 bp immediately flanking it on each side). All cases where the longest match near the sequence was longer than that in the larger surroundings were assumed to be complex tandem duplications, as the expected random fraction of such cases is 1%. We applied the same technique to classify insertions as matching the reverse strand.

### Detecting nucleotide and nucleotide pair preferences

To compute the sequence context preferences of insertion and deletions, we grouped similar events by their type (length, insertion/deletion, and lineage). We also computed the GC content in a window of 400 bp around the event and grouped together events with similar GC content (using bins of 10%). We constructed the nucleotide profile for each event group by simple counting, and transformed the frequency at each position to log odds by comparing it to the background probability in sequences with similar GC content. To detect statistically significant correlations between pairs of positions relative to the insertion or deletion junction, we constructed the joint distributions of nucleotides for each pair of positions in the range -20 to +20 bp relative to insertions or deletions of a given type. We also computed the background joint distribution of nucleotide pairs at each distance. We used chi-square statistics to test if the two contingency tables differ, and a hyper-geometric test to check if particular pairs of nucleotides are correlated in a positive or negative way.

### The indel propensity model

To model the sequence around indels of specific type (length, lineage, insertion/deletion), we constructed a positional Markov model that determines the probability of observing a nucleotide X at position i relative to the indel point by looking up a conditional probability table that is parameterized by the position itself and by the nucleotide at position 'i - 1' (for inserts) and by both the nucleotides at positions 'i - 1' at 'i - l' for deletions (l being the length of the event). The probability tables were inferred directly from the sequences in our compendium. To score a genomic locus for a certain event type, we computed the model likelihood and compared it to the likelihood of a background model that was constructed similarly, but trained using background sequences.

### Exon analysis

To test possible preferences of human exon against high indel propensity, we generated a set of randomized exons by shuffling synonymous codons while preserving GC content. This was done by first determining for each codon its regional GC content and then selecting a synonymous codon at random such that the expected overall GC content distribution at the synonymous sites before and after the randomization is similar. We then computed the fraction of exon loci with indel propensity score larger than 1, in both the real and randomized sets, and determined the significance of the detected differences using binomial statistics.

## Abbreviations

Indel, insertion or deletion.

## Authors' contributions

AT and EDS designed and performed the analysis. AT and EDS wrote the paper.

## Additional data files

The following additional data are available with the online version of this paper. Additional data file 1 is a pdf file including figures S1-S5.

## Supplementary Material

Additional data file 1 Figure S1: length distributions for retained and questionable indels. Shown are the length distributions for insertions and deletions for which no match was found in a direct genomic search (retained events; see Materials and methods) and for insertions and deletions for which the insert or deletion point and its flanking sequence could be matched in the orthologous genome without gaps, contradicting the multiple alignment (questionable events; see Materials and methods). The length distribution is computed separately for each class of events. The overall numbers of retained and questionable indels is given in Figure 1b. Figure S2: chimp sequence quality around loci aligned against human insertions. The distribution of quality scores (higher is better) for the chimp sequence for 20 bp flanks surrounding loci with a human insert with a tandem match, compared to inserts with no match. There is no evidence that the non-tandem events are correlated with low quality sequences. Figure S3: sequence preferences around chimp insertions (see Figure 3 for details). Figure S4: sequence preferences around chimp deletions (see Figure 5 for details). Figure S5: predicting insertion and deletion rates using the indel propensity probabilistic score - chimp results (see Figure 6 for details). Click here for file
