# Supplementary material for: Sequence context affects the rate of short insertions and deletions in flies and primates
Source: Genome Biol. 2008 Feb 21;9(2):R37. doi: 10.1186/gb-2008-9-2-r37 (PMC2374710; doi:10.1186/gb-2008-9-2-r37)
Supplement: Additional data file 1 — Figure S1: length distributions for retained and questionable indels. Shown are the length distributions for insertions and deletions for which no match was found in a direct genomic search (retained events; see Materials and methods) and for insertions and deletions for which the insert or deletion point and its flanking sequence could be matched in the orthologous genome without gaps, contradicting the multiple alignment (questionable events; see Materials and methods). The length distribution is computed separately for each class of events. The overall numbers of retained and questionable indels is given in Figure 1b. Figure S2: chimp sequence quality around loci aligned against human insertions. The distribution of quality scores (higher is better) for the chimp sequence for 20 bp flanks surrounding loci with a human insert with a tandem match, compared to inserts with no match. There is no evidence that the non-tandem events are correlated with low quality sequences. Figure S3: sequence preferences around chimp insertions (see Figure 3 for details). Figure S4: sequence preferences around chimp deletions (see Figure 5 for details). Figure S5: predicting insertion and deletion rates using the indel propensity probabilistic score - chimp results (see Figure 6 for details). [file gb-2008-9-2-r37-S1.pdf]

Sup Fig 1

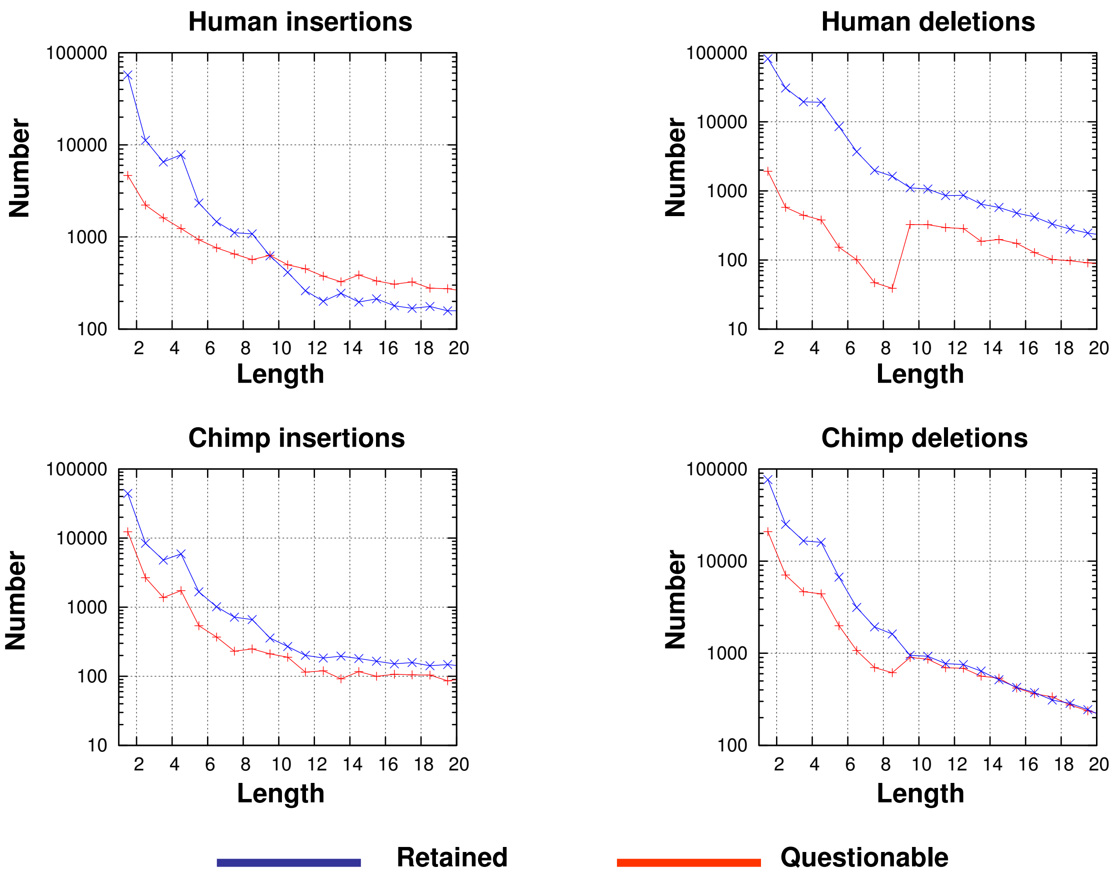

Sup Fig 2

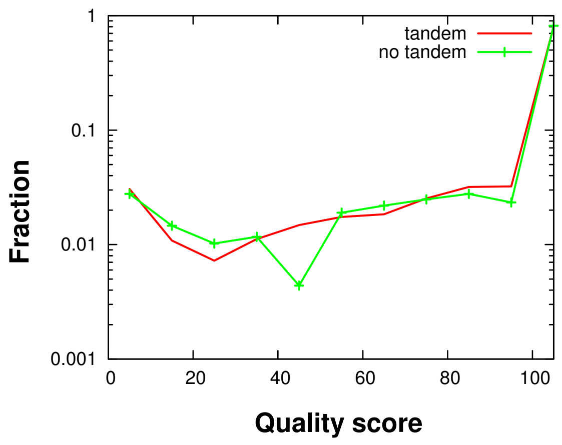

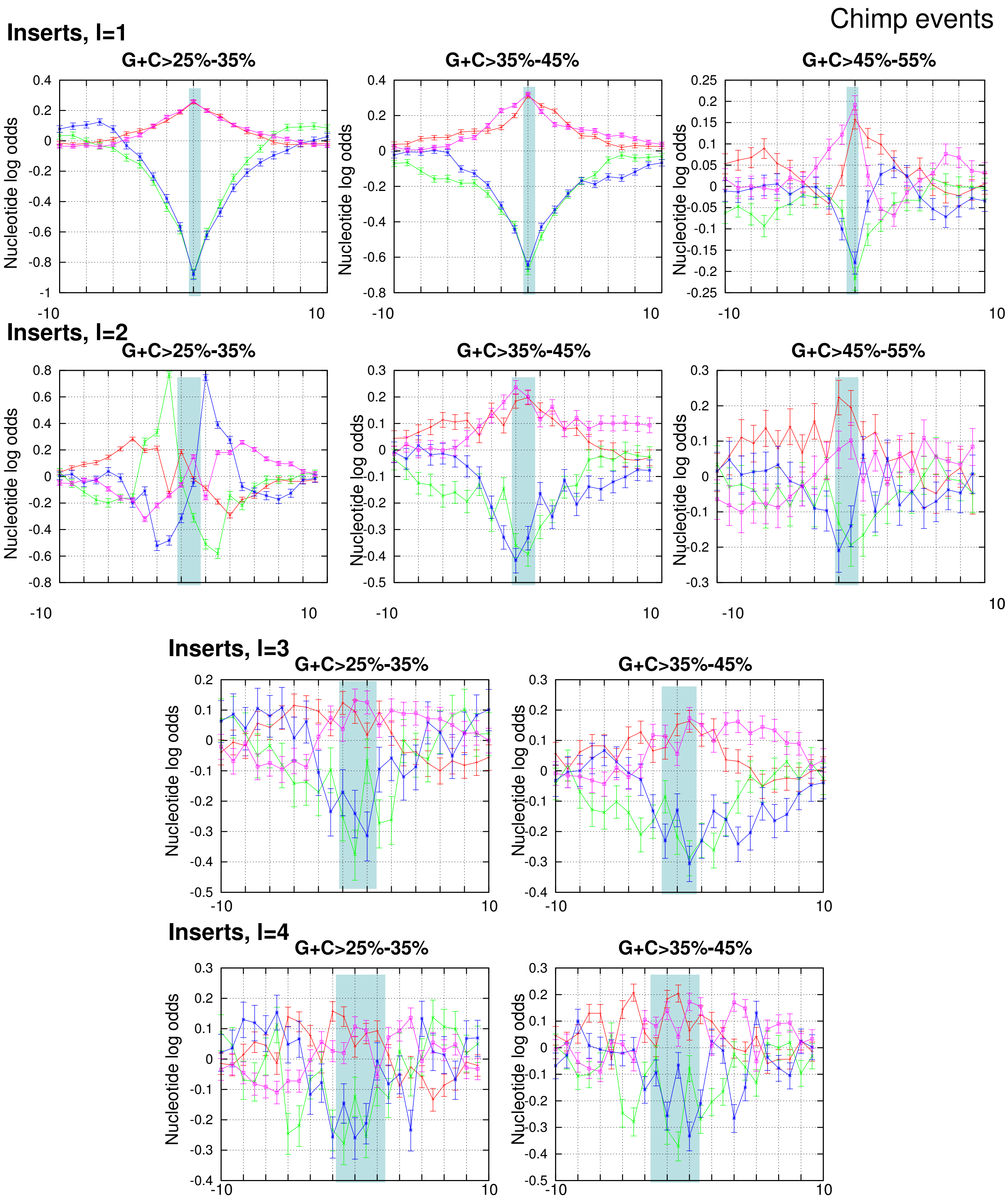
Chimp events
Inserts, l=1
G+C>25%-35%
G+C>35%-45%
G+C>45%-55%
Inserts, l=2
G+C>25%-35%
G+C>35%-45%
G+C>45%-55%
Inserts, l=3
G+C>25%-35%
G+C>35%-45%
Inserts, l=4
G+C>25%-35%
G+C>35%-45%
Nucleotide log odds
-10
10

Sup Figure 3

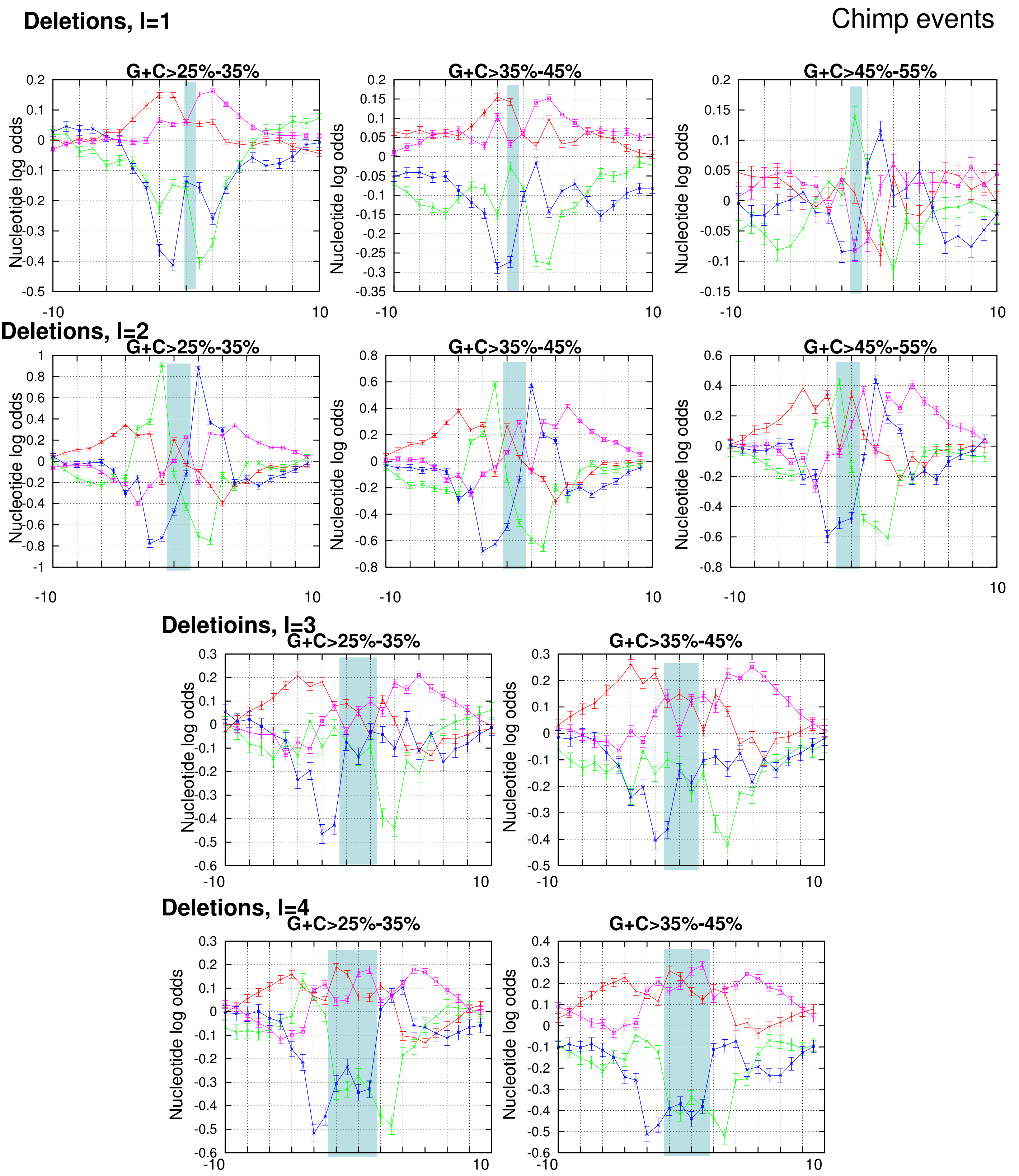
Deletions, l=1
Chimp events
G+C>25%-35%
G+C>35%-45%
G+C>45%-55%
Deletions, l=2
G+C>25%-35%
G+C>35%-45%
G+C>45%-55%
Deletioins, l=3
G+C>25%-35%
G+C>35%-45%
Deletions, l=4
G+C>25%-35%
G+C>35%-45%
Nucleotide log odds
-10
10

Sup Figure 4

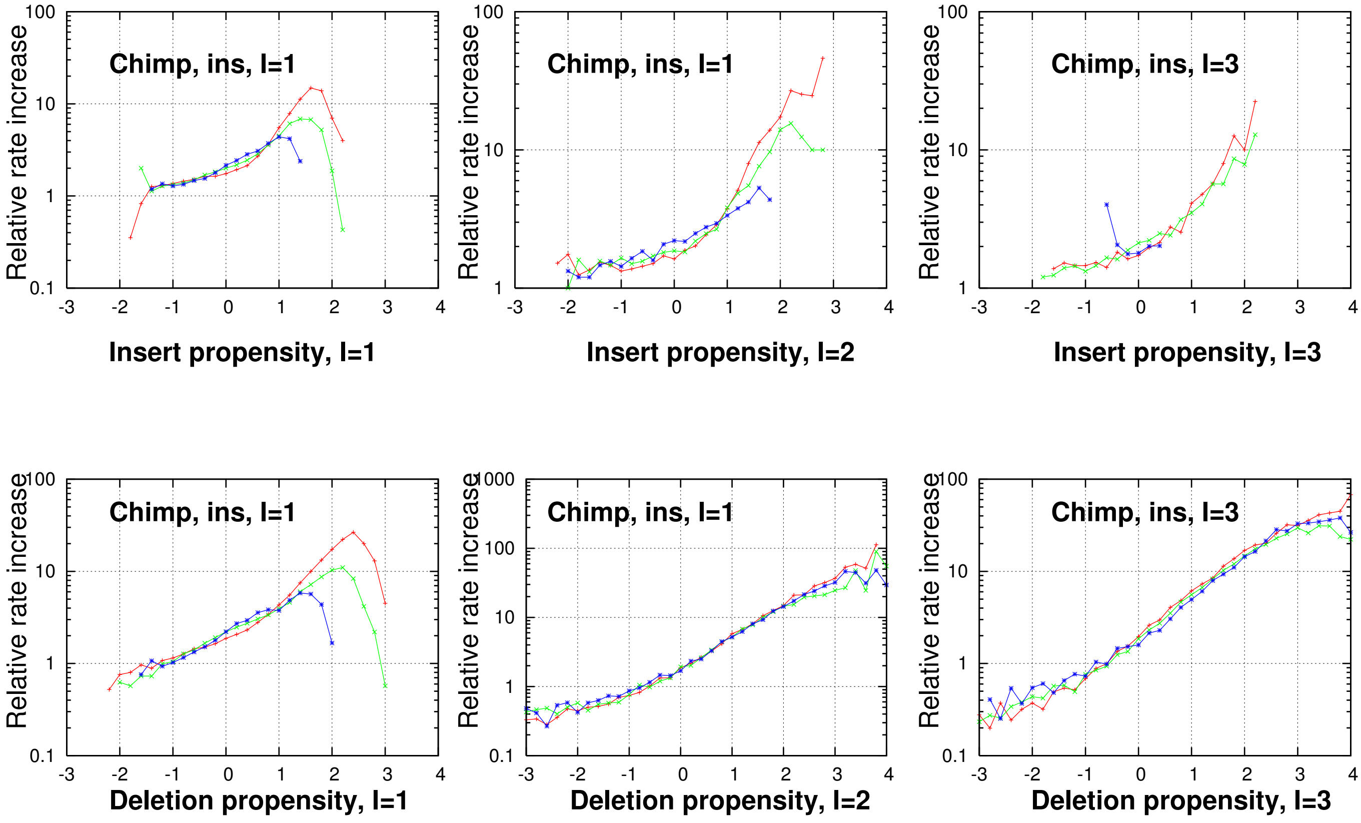
Chimp, ins, l=1
Chimp, ins, l=1
Chimp, ins, l=3
Relative rate increase
Insert propensity, l=1
Insert propensity, l=2
Insert propensity, l=3
Chimp, ins, l=1
Chimp, ins, l=1
Chimp, ins, l=3
Deletion propensity, l=1
Deletion propensity, l=2
Deletion propensity, l=3
0.1
1
10
100
1000
-3
-2
-1
0
1
2
3
4

Sup Figure 5
